# American Medical Association

**Published:** 1853-09

**Authors:** 


### ART. IV.—*American Medical Association. Its Volumes of Transactions.*

In the August number of this Journal, a correspondent indulges in some complaints against the American Medical Association, charging it with having failed to meet the expectations of some of its friends—or otherwise the medical profession; and gives utterance to some gloomy forebodings of its future success, predicting a speedy dissolution; an end which, if consummated, I fear will be far from glorious to the medical profession of the United States.

Never having been within the portals of this vast esculapian temple, and having never mingled in the deliberations of the Solons assembled in this Hippocratian congress, and being led to believe from the published reports, and verbal communications of those who have from time to time mingled in the councils of our medical sages, that all was peace and quietness within, and that they found it pleasant and profitable to be together, I must confess I read with no little surprise and pain the history of the want of success in this association, and the sad prognosis of its future continuance, as detailed by your correspondent.

Notwithstanding all that he has said, I can but believe, despite of all its shortcomings, and disappointments to some of its friends, that it has accomplished not a little that is really good, and of a beneficial character to the profession in our country; nor dismiss the hope that its existence will be perpetuated, and cease only with our nationality.

I wish to call attention to one point in the communication referred to. The writer complains of the want of interest manifested in the association by the profession at large, and among other things, evidences as proof, the limited number of copies of the Transactions circulated among medical men; that but very few copies are taken by physicians except those which are subscribed for by the members, "who are as a matter of course expected to purchase a copy."

This is certainly an evil, and just as certainly capable of a remedy. Doubtless the utility of the association would be greatly extended, and the interest in its prosperity and popularity, among the great body of the profession enenhanced and retained, by a circulation of these volumes of Transactions co-extensive with the ranks of that profession.

I would respectfully submit to the association, (and by the association I mean its members individually as well as collectively,) that an effort to increase the facilities by which access to these Transactions may be had, is worthy of their consideration.

In the first place, the price of these volumes is too high to place them within reach of all who may wish to obtain them. This high price is occasioned by the limited number printed. A number barely sufficient to pay for the publishing is issued, and there the matter ends. It is now almost impossible to obtain a set of the back volumes at any price. A little financiering upon the part of somebody might have obviated this difficulty in the past, and certainly can in the future.

Then again the process of obtaining them is too difficult, and requires a routine of correspondence, mail carrying, and postage paying, involving a further tax of time and money.

These volumes should be published in larger numbers, and consequently at a cheaper rate; and placed in the book-stores, within the ability and reach of every person desiring to purchase.

I have just seen the notice of the forthcoming volume, given by the treasurer, in which the price of a single volume is fixed at five dollars. True, by taking half a dozen copies you can obtain them for a few shillings less, and if twenty-five copies are taken seventy-five dollars will pay for them.

But to save these two dollars upon the price of the book, some dollar-loving, or some dollar-needy individual is obliged to canvass his district, be the territory great or small, to obtain for others a favor equal to that which he himself receives, and have his labor thrown into his part of the bargain.

It is stated in the above notice, that "at least fifteen hundred dollars, in addition to the amount already received by the treasurer, will be required to defray the cost of publication."

After this amount of money is raised, and the requisite number printed to fulfill the terms of the contract in raising the same, I presume that as in former cases the press will stop, and the supply cease.

Why must there be all this waste of typesetting, and of the weary toil in preparing these limited number of volumes? Can it not be preserved by stereotyping for future use? Or, cannot the work at this stage be disposed of to some publisher on a speculation, who will give us cheap copies, and fill the shelves of our booksellers with them? Are not these volumes of value and interest sufficient to enable the association to dispose of its interest in them at the outset, have them got up under their supervision, and distributed to the profession as are all other new and valuable publications? Publishers find no difficulty in causing other works in which they are peculiarly interested, to penetrate the remotest bounds of the profession.

If the association must at any rate father these works in their incipiency, is there not magnanimity enough among our medical Astors and Girards, to secure, not only as now, the printing of a limited number, but devise some means by which the copies may be greatly multiplied?

This subject would seem certainly to be of importance enough to demand the attention of the association, and subtract a little from the time usually given to constitution mending. We can hardly expect a very deep interest to be felt in its welfare, when not one in fifty of the profession are able to obtain a knowledge of its doings.

An outsider would suggest that the debility and signs of early decay, discovered so soon in our patient, may not *all* be *constitutional*, but perchance may be in part at least, the result of too early and too close an application of the mind and energies to *one object*, and to *one pursuit*, and that renewed vigor may be infused, by removal to a *purer air*, a *field of greater size and more extensive cultivation.*

BUFFALO, August, 1853. J. M. N.

